# *Lactobacillus reuteri* DSM 17938 and Its Supernatant Ameliorate Parkinson’s Disease in Association with Modulation of Gut Microbiota and Its Tryptophan Metabolism

**DOI:** 10.3390/antiox15070882

**Published:** 2026-07-16

**Authors:** Bin Su, Fanying Meng, Yao Lu, Yunlong Zhang, Tingting Wang, Yuxin Zhang, Jiajia Liu, Lianbing Lin

**Affiliations:** 1 Faculty of Life Science and Technology, Kunming University of Science and Technology, Kunming 650500, China; subin_kust_ynmzu@163.com (B.S.); 1026792964@qq.com (F.M.); 2639484499@qq.com (Y.L.); 15974849114@163.com (Y.Z.); 1498354106@qq.com (T.W.); 921024546@qq.com (Y.Z.); liujiajialjj@163.com (J.L.); 2 Engineering Research Center for Replacement Technology of Feed Antibiotics of Yunnan College, Kunming 650500, China

**Keywords:** Parkinson’s disease, gut microbiota, *Lactobacillus reuteri* DSM 17938, microbial tryptophan metabolites, probiotics

## Abstract

Parkinson’s disease (PD) is a neurodegenerative disorder associated with gut dysbiosis and tryptophan metabolism disturbance, but the mechanisms of probiotic action are unclear. We orally administered *Lactobacillus reuteri* DSM 17938 live bacteria (1.0 × 10^9^ CFU/mL, 0.2 mL) or its fermentation supernatant lyophilized powder at three concentrations to MPTP-induced PD male C57BL/6J mice. Treatments increased locomotor distance and speed, elevated serum SOD and GSH, reduced MDA, TNF-α, IL-6, and IL-1β, promoted neuronal survival, and tended to increase TH expression in the substantia nigra. 16S rRNA sequencing showed that treatments altered gut microbiota composition. PD mice had increased *Lactobacillus* and *Allobaculum* but decreased *Oscillospira* and *Helicobacter*; treatments restored *Oscillospira*. Fecal untargeted metabolomics revealed disturbed tryptophan metabolism in PD, with elevated indoleacetic acid and reduced kynurenic acid, xanthurenic acid, indole-3-ethanol, and α-oxo-1H-indole-3-propanoic acid. Treatments significantly restored neuroprotective metabolites including kynurenic acid, xanthurenic acid, and serotonin. PICRUSt2 predicted tryptophan synthesis pathway-associated microbes (*Oscillospira*, *Ruminococcus*, *Coprococcus*). Treatments ameliorated motor deficits, oxidative stress, inflammation, and dopaminergic neuron death, correlating with gut microbiota modulation and accumulation of microbiota-derived tryptophan metabolites. Live bacteria showed superior antioxidant and neuroprotective efficacy compared to supernatant, likely due to sustained colonization and continuous metabolic activity. These findings suggest that targeting key tryptophan-metabolizing bacteria or their metabolites may be a potential PD therapy.

## 1. Introduction

Parkinson’s disease (PD) is the world’s second-leading neurodegenerative disorder. It features an insidious onset and progressive nature, which complicates early clinical detection and imposes a substantial and growing socioeconomic burden [1]. Pathologically, PD exhibits progressive degeneration of dopaminergic neurons and reduced tyrosine hydroxylase (TH) expression in the substantia nigra (SN), leading to motor impairments such as resting tremor, gait disturbance, bradykinesia, and rigidity [2]. The pathogenesis of PD involves a complex interaction of genetic predisposition, oxidative stress, metabolic disturbances, environmental factors, and neuroinflammation [3,4]. Moreover, emerging evidence has highlighted the role of the gut microbiome in PD [5]. Accumulating evidence points to a contribution of gut microbiota dysbiosis and altered metabolic patterns to PD, likely mediated by a dysregulated gut–brain axis that affects both central and enteric nervous systems [6]. Based on this, extensive research has investigated the relationship between gut microbiota and neurodegenerative diseases. For instance, a study has revealed that PD patients exhibit an increase in the abundance of *Catabacter*, *Akkermansia*, and *Akkermansiaceae*, while reducing *Faecalibacterium*, *Roseburia*, and *Lachnospiraceae* [7].

Probiotics refer to living microbes that deliver health-promoting effects to the host. Targeted modulation of the gut microbiota through probiotic supplementation exhibits considerable potential for the treatment of PD [8]. *Lactobacillus reuteri* is a gut commensal capable of colonizing the intestinal tracts of humans and other animals, withstanding a wide variety of pH environments, and inhibiting pathogenic microorganisms [9]. The health benefits of postbiotics primarily derive from microbial metabolites and cellular components, encompassing inactivated whole cells, extracellular vesicles, and secreted metabolites [10]. Research evidence suggests that gamma-aminobutyric acid (GABA) derived from *Lactobacillus reuteri* can alleviate MPTP-induced PD by inhibiting ferroptosis through the AKT/GSK3β/GPX4 signaling axis [11]. By regulating tryptophan catabolism, *Lactobacillus reuteri* DSM 17938 exerts a neuroprotective effect on spinal cord injury. This regulation activates AHR-dependent signaling, with consequent inhibition of M1 microglial responses and alleviation of local neuroinflammation, thereby enhancing recovery outcomes [12]. Recent research increasingly recognizes the important role played by the gut microbiota in regulating central neurochemical pathways through the gut–brain axis (GBA) [13]. *Lactobacillus reuteri* DSM 17938 reinforces the gut barrier via tight-junction up-regulation, prevents neurotoxin entry, and reverses permeability and tight-junction protein defects in mouse intestinal injury models [14], while other strains have been demonstrated to increase the expression of ZO-1 and occludin through the MLCK pathway [15].

Tryptophan metabolic dysregulation is a common feature in PD patients [16]. Alterations in the intestinal tryptophan metabolic profile are highly interrelated with changes in the gut microbiota [17]. Tryptophan constitutes an indispensable aromatic amino acid and possesses the highest molecular weight among all proteinogenic amino acids. While tryptophan is the scarcest constituent within cells and proteins, it nevertheless functions as a critical biosynthetic intermediate for diverse microbial metabolites [18]. Within the gut, the vast majority of tryptophan (about 95%) is channeled through the kynurenine route to produce kynurenine, kynurenic acid, xanthurenic acid, quinolinic acid, and picolinic acid. Only a small fraction (1–2%) follows the alternative pathway leading to serotonin and melatonin [19]. The metabolites of the kynurenine pathway exhibit divergent neuroactivities: for example, kynurenine, kynurenic acid, and xanthurenic acid are neuroprotective, whereas others, such as 3-hydroxykynurenine, 3-hydroxyanthranilic acid, and quinolinic acid, are neurotoxic [20,21]. Substantial evidence has confirmed the impairment of the kynurenine pathway in individuals with PD [22,23]. There is research suggesting that promoting the accumulation of kynurenine pathway metabolites, which have neuroprotective effects, represents a considerable potential strategy for PD [24].

Although probiotic intervention targeting the gut microbiota and its tryptophan metabolic pathways holds promise for alleviating PD, the molecular basis by which *Lactobacillus reuteri* DSM 17938 regulates gut microbiota and tryptophan metabolism to ameliorate PD remains incompletely understood. Moreover, the relative protective efficacy of live bacteria versus its supernatant in PD models has not been systematically compared. Therefore, this study aimed to evaluate the protective effects of live *Lactobacillus reuteri* DSM 17938 and its fermentation supernatant at three different concentrations in MPTP-induced PD mice, and to explore whether these effects are associated with alterations in gut microbiota composition and tryptophan metabolite profiles using 16S rRNA sequencing and fecal untargeted metabolomics. We hypothesized that both live bacteria and supernatant treatments may confer neuroprotection through the modulation of specific tryptophan-metabolizing microbes and their related metabolites, with live bacteria potentially exhibiting superior efficacy due to sustained gut colonization. By integrating behavioral assessments, serum biochemical parameters, histopathological analyses, and multi-omics data, this study sought to provide novel experimental evidence for PD intervention strategies targeting the microbiota-tryptophan metabolism axis.

## 2. Methods

### 2.1. The Preparation of Lactobacillus reuteri DSM 17938 or Its Supernatant

*Lactobacillus reuteri* DSM 17938 was cultured in MRS broth (Guangdong Huankai Technology Co., Ltd., Foshan, China). A seed culture of Lactobacillus reuteri DSM 17938 was prepared from a cryopreserved stock by inoculation into 250 mL of MRS broth and incubation at 37 °C for 24 h. This seed culture was then transferred as a 5% (*v*/*v*) inoculum into 3 L of fresh MRS broth and incubated under the same conditions for scale-up cultivation. During fermentation, the pH decreased from 5.6 to 4.1. After centrifugation at 10,000× *g* for 5 min at 4 °C, the supernatant was removed, and the pellet was washed with PBS and then re-collected by centrifugation. The collected cells were adjusted to 1.0 × 10^9^ CFU/mL; this suspension was designated as the YLB treatment. For supernatant preparation, 200 mL of the supernatant was lyophilized for 72–96 h, and the crude supernatant was used without pH neutralization or subsequent filtration to preserve the complete metabolite profile, in line with the aim of evaluating the holistic bioactivity of the fermentation metabolites. Powder was ground, packaged, and stored at −80 °C until use. The lyophilized powder (equivalent to 200 mL of original supernatant) was reconstituted in 20 mL of sterile water to obtain a 10×concentrated stock solution. This stock solution was then diluted with sterile water to yield three concentrations: YCFS1 (1:10 dilution, from 1 mL stock + 9 mL water), YCFS3 (1:4 dilution, from 3 mL stock + 9 mL water), and YCFS5 (1:2 dilution, from 3 mL stock + 3 mL water). These represented low, medium, and high concentrations of supernatant, respectively.

### 2.2. Animal Groups and Experimental Design

**Figure 1:**
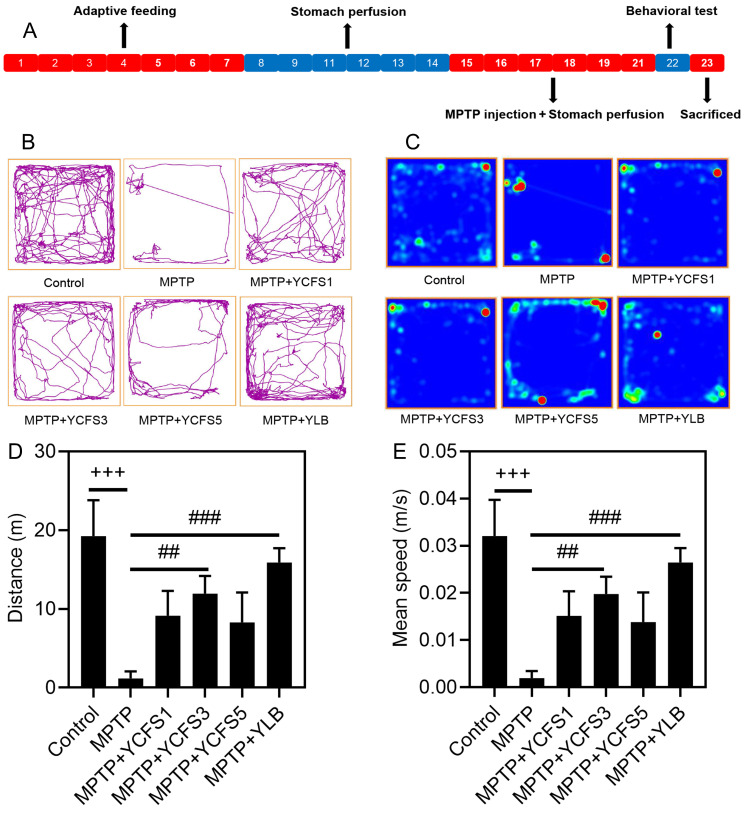
*Lactobacillus reuteri* DSM 17938 or its supernatant ameliorates motor deficits in PD mice, with n = 3 biological replicates per group. (**A**) Experimental procedure timeline. (**B**) Representative movement trajectories in the open field test. (**C**) Heat maps showing the spatial distribution of locomotor activity; red indicates areas where mice exhibited prolonged dwell time. (**D**) Total distance traveled (m). (**E**) Mean speed (m/s). Data are expressed as means ± SEM and analyzed via one-way ANOVA. Where the +++ represents that the difference between MPTP and control was significant, and *p* < 0.001. The ## and ### represents that the difference between MPTP + YCFS1, MPTP + YCFS3, MPTP + YCFS5, and MPTP + YLB with MPTP was significant, and ##: *p* < 0.01, ###: *p* < 0.001; nonsignificant differences were not labeled. Data are expressed as means ± SEM and analyzed via one-way ANOVA.

Male C57BL/6J mice aged six weeks (body weighing 18–21 g) were acquired from SPF (Beijing) Biotechnology Co., Ltd., Beijing, China. and housed in a specific pathogen-free room at the experimental animal center of Kunming University of Science and Technology. The room was kept under standard conditions (22 ± 2 °C, 50–60% humidity, 12 h light/12 h dark cycle), with free access to food and water. Mice were divided into 6 groups after adaptive feeding for one week. A sample size of 10 mice per group was set based on previous studies [11,25]. MPTP-HCl (Shanghai Beyotime Biotech Inc., Shanghai, China SH3695) was dissolved in sterile 0.9% saline and administered via intraperitoneal injection at a dose of 30 mg/kg body weight once daily for 8 consecutive days (day 14–21, subacute regimen). Control group: 7–21 day gavage with 0.9% NaCl (0.2 mL per day at 9:00 PM), 14–21 day intraperitoneal injection 0.9% NaCl (0.2 mL per day at 9:00 AM). MPTP group: 7–21 day gavage with of 0.9% NaCl (0.2 mL per day at 9:00 PM), 14–21 day intraperitoneal injection of 30 mg/kg MPTP (0.2 mL per day at 9:00 AM). MPTP + YCFS1 group: 7–21 day gavage with YCFS1 (0.2 mL per day at 9:00 PM), 14–21 day intraperitoneal injection of 30 mg/kg MPTP (0.2 mL per day at 9:00 AM). MPTP + YCFS3 group: 7–21 day gavage with YCFS3 (0.2 mL per day at 9:00 PM), 14–21 day intraperitoneal injection of 30 mg/kg MPTP (0.2 mL per day at 9:00 AM). MPTP + YCFS5 group: 7–21 day gavage with of YCFS5 (0.2 mL per day at 9:00 PM), 14–21 day intraperitoneal injection of 30 mg/kg MPTP (0.2 mL per day at 9:00 AM). MPTP + YLB group: 7–21 day gavage with of *Lactobacillus reuteri* DSM 17938 (0.2 mL, 1.0 × 10^9^ CFU/mL, per day at 9:00 PM), 14–21 day intraperitoneal injection of 30 mg/kg MPTP (0.2 mL per day at 9:00 AM). And the timeline in this study was shown in Figure 1A.

### 2.3. Animal Behavioral Test

We used the open field test to evaluate motor function in all groups on day 22. The test was performed in a square chamber (400mm × 400mm × 300 mm). Each mouse was placed in the chamber, and its movement was recorded for 10 min by an overhead camera. After all tests were completed, the videos were given random codes. We then used the AnyMaze 7.54 software to analyze these videos for the total distance traveled, movement paths, and heat maps. The chamber was wiped with 75% ethanol before each test to remove any lingering smells. To minimize observer bias, the open field test was conducted by dedicated personnel fully blinded to group allocation. The testing order was randomly determined prior to the experiment. All videos were analyzed using AnyMaze software by an independent investigator who remained blinded throughout the entire process. Statistical analyses were completed prior to unblinding. Values are expressed as mean ± standard error of the mean (SEM). All groups were compared using one-way ANOVA followed by Dunnett’s using GraphPad Prism 8.

### 2.4. Histology Evaluation

On day 23, immediately after fecal sample collection, mice were anesthetized with tribromoethanol (400 mg/kg), blood was collected via cardiac puncture, and mice were subsequently euthanized by cervical dislocation. Brain sections from the same 3 mice per group that were used for the open field test were collected for histological analysis. For HE staining, sections were deparaffinized in eco-friendly clearing agent (Wuhan Servicebio Technology Co., Ltd., Wuhan, China) for 40 min, rehydrated, stained with hematoxylin for 5 min, differentiated, blued, counterstained with eosin for 15 s, dehydrated, cleared in n-butanol and xylene, and mounted; for Nissl staining, sections were stained in Nissl solution for 5 min, rinsed, differentiated briefly in 0.1% glacial acetic acid, dried, cleared in xylene, and coverslipped, with all images captured using a Nikon microscope (Eclipse C1). For immunofluorescence, deparaffinized sections underwent antigen retrieval in citrate buffer (pH 6.0) via microwave heating (medium power 8 min, stand 8 min, medium-low 7 min), then cooled, washed with PBS, blocked with 3% BSA for 30 min, and incubated with primary antibody (TH, 1:1000, Servicebio) overnight at 4 °C; after PBS washes, sections were incubated with CY3-conjugated goat anti-rabbit secondary antibody (1:300, Servicebio) for 50 min at room temperature in the dark, followed by DAPI (Servicebio) for 10 min, then treated with autofluorescence quencher B (Servicebio) for 5 min, washed, and mounted, with imaging performed using an upright fluorescence microscope (Nikon Eclipse C1) and a slide scanner (Pannoramic MIDI). For immunohistochemistry, deparaffinized and antigen-retrieved sections were treated with 3% H_2_O_2_ for 25 min in the dark to block endogenous peroxidase, blocked with 3% BSA for 30 min, incubated with primary antibody (1:1000 in PBS) overnight at 4 °C, followed by HRP-conjugated goat anti-rabbit IgG (1:200, Servicebio) for 50 min at room temperature; signal was developed with DAB substrate, monitored microscopically, stopped with tap water, counterstained with hematoxylin for 3 min, differentiated, blued, dehydrated and cleared through graded ethanol, n-butanol, and xylene, mounted, and imaged using a Nikon microscope. The TH-positive areas were quantified and statistically analyzed using ImageSXM software. All data are expressed as mean ± standard error of the mean (SEM), and group comparisons were performed using one-way ANOVA followed by Dunnett’s post hoc test with GraphPad Prism 8.

### 2.5. Serum Antioxidant and Anti-Inflammatory Parameters

On day 23, immediately after fecal sample collection, blood was collected via cardiac puncture from mice under tribromoethanol anesthesia (400 mg/kg), prior to euthanasia by cervical dislocation, for serum analysis of inflammatory cytokines interleukin-6 (IL-6), interleukin-1β (IL-1β), and tumor necrosis factor-α (TNF-α), and oxidative stress markers superoxide dismutase (SOD), malondialdehyde (MDA), and glutathione (GSH) were measured using ELISA kits (Servicebio Technology Co., Ltd, Wuhan, China; SOD: GM1133, GSH: GM1135, MDA: GM1134, TNF-α: GZEM0004-48T, IL-6: GZEH0001-48T, IL-1β: GEM0002-48T) according to the manufacturers’ protocols. Data are expressed as mean ± standard error of the mean (SEM), and all groups were compared using one-way ANOVA followed by Dunnett’s test with GraphPad Prism 8.

### 2.6. Fecal Sample Collection

Fecal samples were collected on day 23 following the completion of behavioral testing, and importantly, prior to euthanasia and tissue harvesting. Mice were individually placed into sterile, empty cages, each lined with a sterilized filter paper, and allowed to defecate spontaneously. Fecal pellets were collected immediately upon completion of defecation into sterile microcentrifuge tubes. After being flash-frozen in liquid nitrogen, the samples were maintained at –80 °C for later use. Immediately after fecal collection, the same cohort of mice was anesthetized for blood collection and subsequent euthanasia, followed by brain tissue removal (as described in Section 2.4 and Section 2.5).

### 2.7. DNA Extraction for Fecal and 16S rRNA Gene Amplicon Generation and Bioinformatic Analysis

An aliquot of 0.5 g was transferred into an EP tube, followed by the addition of the lysis buffer for extraction and subsequent homogenization. The Omega Soil DNA Kit (Norcross, GA, USA) was used to extract nucleic acids from the ground homogenate. Quantification of the extracted DNA was performed via NanoDrop UltraFL, whereas its molecular size was assessed by electrophoresis on a 0.8% agarose gel. For bacterial 16S rRNA gene analysis, the hypervariable V3–V4 region (spanning roughly 480 bp) is routinely targeted as the amplicon for sequencing. For PCR amplification, the specific primers were 338F (5′-barcode + ACTCCTACGGGAGGCAGCA-3′) and 806R (5′-GGACTACHVGGGTWTCTAAT-3′). The amplification system consisted of 0.25 μL of ABclonal DNA polymerase, 5 μL of 5 reaction buffer, 2 μL of dNTP (10 mM), 1 μL of reverse primer (10 μM), 5 μL of 5 High GC Buffer, 2 μL of template DNA, 8.75 μL of water, and 1 μL of forward primer (10 μM). The PCR protocol was set as follows. An initial denaturation step at 98 °C lasted for 5 min. This was followed by 25 amplification cycles, each consisting of 98 °C for 30 s, 52 °C for 30 s, and 72 °C for 45 s. Upon completion, a final extension was conducted at 72 °C for 5 min, after which the reaction was held indefinitely at 12 °C. Finally, the target DNA fragments were purified via 2% agarose gel electrophoresis, followed by size selection with magnetic beads. The PCR products were first quantified using the Quant-iT PicoGreen dsDNA assay kit (Carlsbad, CA, USA) on a microplate reader (BioTek Instruments, Inc., Winooski, VT, USA, FLx800). Subsequently, equal amounts of each product were pooled into a single sample for downstream testing. Illumina’s TruSeq Nano DNA LT library prep kit (Illumina, Inc., San Diego, CA, USA) was employed for constructing the sequencing libraries. The library was finally subjected to fragment selection and purification by 2% agarose gel electrophoresis. 1 μL of the library was taken; first, it was quality-controlled on an Agilent Bioanalyzer 2100 (Agilent Technologies, Santa Clara, CA, USA) using the Agilent high sensitivity DNA kit (Agilent Technologies, Santa Clara, CA, USA), and secondly, it was quantified on a Promega Quanti Fluor (Promega Corporation, Madison, WI, USA) using the Quant-iT PicoGreen dsDNA assay kit (Carlsbad, CA, USA). Qualified libraries (concentration > 2 nM) from each group were subjected to paired-end sequencing on an Illumina NovaSeq (Illumina, San Diego, CA, USA). Raw paired-end sequencing reads were processed via QIIME2 (version 2023.9) using the DADA2 plugin (v1.20.0) for quality denoising, paired-end merging, and chimera removal to resolve amplicon sequence variants (ASVs) [26], with forward reads truncated to 240 bp and reverse reads to 200 bp based on per-base quality profiles, and quality filtering parameters set as maxEE = 2.0 and trunc Q = 2. Rarefaction curves were generated to confirm sequencing saturation, and all samples were rarefied to 10,000 clean reads per sample for alpha and beta diversity analyses. Beta diversity was assessed using the Bray–Curtis distance metric, visualized via NMDS, and PERMANOVA was performed with 999 permutations (*p* < 0.05). Taxonomic assignment of ASVs was performed using a Naive Bayes classifier trained against the Greengenes (13-8) 99% reference sequences, and genus-level relative abundance was directly summarized based on the taxonomic assignments of ASVs. Differential abundance of microbial genera was analyzed using ANCOM-BC with FDR correction (q < 0.05). Functional potential of the gut microbiome was predicted using PICRUSt2 (Phylogenetic Investigation of Communities by Reconstruction of Unobserved States, version 2.5.2) based on the ASV table and representative sequences. Briefly, ASV sequences were placed into reference phylogenetic trees using the EPA-NG algorithm, and gene family abundances were inferred via the hsp method. These gene family abundances were then collapsed to MetaCyc pathway abundances to generate predicted metabolic profiles, providing genomic-level insights into the metabolic capabilities of the gut microbial community. Differentially enriched pathways between groups were identified using Welch’s *t*-test, with FDR-adjusted q < 0.05 considered statistically significant. The 16S rRNA gene data were analyzed using QIIME2 (version 2023.9) software and the Greengenes database. Potential functional profiling of the microbiota was conducted using PICRUSt2 [27].

### 2.8. Fecal Untargeted Metabolomics Analysis

A 50 mg fecal sample was weighed into a 2 mL centrifuge tube, followed by the addition of 500 μL of methanol containing 5 ppm 2-chlorophenylalanine (pre-chilled) and two steel beads. The mixture was vortexed for 30 s and then homogenized using a high-throughput tissue grinder (Scientz-192, Ningbo Scientz Biotechnology Co., Ltd., Ningbo, China) at 55 Hz for 60 s, with this grinding step repeated twice. Subsequently, the sample was placed in an ultrasonic cleaner (KQ-200KDE, Kunshan Ultrasonic Instrument Co., Ltd., Kunshan, China) for 10 min and then in a −20 °C freezer for 30 min. After centrifugation at 12,000 rpm for 10 min (4 °C), the supernatant was collected and filtered through a 0.22 μm membrane for analysis. Separation was performed on an ACQUITY UPLC HSS T3 column (100 Å, 1.8 μm, 2.1 mm × 100 mm) with a flow rate of 0.4 mL/min, column temperature maintained at 40 °C, autosampler temperature at 8 °C, and an injection volume of 2 μL. The mobile phases for both positive and negative modes consisted of solvent A (0.1% formic acid in water) and solvent B (acetonitrile containing 0.1% formic acid). A Thermo Orbitrap Exploris 120 mass spectrometer (Thermo Fisher Scientific, Inc., Waltham, MA, USA) controlled by Xcalibur software (v4.7, Thermo) was used to acquire DDA mass spectrometry data in both positive and negative ion modes. The raw data were imported into Compound Discoverer™ 3.3 (v3.3.2.31, Thermo, Waltham, MA, USA) for peak extraction, alignment, and correction based on its advanced peak detection and peak quality scoring algorithm. The unique peak rating calculation and filtering significantly reduced interference from background and low-quality peaks. Peaks detected in fewer than 50% of QC samples were excluded, missing values were imputed using the Fill Gaps algorithm, all peak areas were normalized to the total sum, and metabolic features with a coefficient of variation (CV) exceeding 30% across QC samples were subsequently excluded to ensure data reproducibility. Metabolite identification was performed by matching against in-house libraries, the mzCloud online database, LIPID MAPS, HMDB, and the NIST_2020_MSMS spectral library, with an MS1 mass tolerance set to 15 ppm and an MS2 match factor threshold set to 50. It should be noted that, in the absence of authentic chemical standards for all target compounds, the metabolite identifications reported herein are considered putatively annotated (MSI level 2) based on high-resolution MS/MS spectral matching against public databases (mzCloud, HMDB, NIST).

Metabolite identification was performed at Metabolomics Standards Initiative (MSI) level 2 (putatively annotated based on MS/MS spectral matching), as no authentic standards were used for absolute quantification. Differential analysis between two groups was conducted using R4.4.3 software. For each pairwise comparison, the normality of log-transformed peak areas for each metabolite was assessed using the Shapiro–Wilk test (*p* > 0.05 considered normal distribution). For metabolites satisfying the normality assumption, Student’s *t*-test was applied; for those not meeting normality, the Wilcoxon rank-sum test was used. Differential metabolites between groups were identified using a combined filtering strategy. Metabolites with an absolute log2 fold-change (|log_2_FC|) ≥ 1.0 were considered biologically significant. Univariate statistical significance was determined with a nominal *p*-value < 0.05. To control the false discovery rate (FDR) arising from multiple comparisons, the Benjamini–Hochberg (BH) procedure was applied, and metabolites with FDR-adjusted *p*-value < 0.10 were retained. For metabolites selected via PLS-DA models, an additional criterion of Variable Importance in Projection (VIP) score > 1.0 was used as a secondary filter to prioritize metabolites contributing most to group separation. PLS-DA model validation was performed using permutation testing (n = 200 permutations), with a negative Q^2^ intercept indicating no overfitting. Metabolites were considered differentially abundant only when they simultaneously met both the FC and FDR criteria. These criteria were applied consistently to all pairwise comparisons. Functional analysis of differential metabolites was performed primarily through KEGG enrichment analysis using clusterProfiler (v4.6.0) to identify significantly enriched metabolic pathways, capturing the overall change trends of all metabolites within a pathway and facilitating the screening of key pathways.

## 3. Result

### 3.1. Lactobacillus reuteri DSM 17938 or Its Supernatant Ameliorate the Motor Deficits of MPTP-Induced PD Mice

The open-field test was employed to assess locomotor performance across all experimental mouse groups. Trajectory plots and heatmap analysis showed that control mice were highly active, exhibiting dense and complex movement patterns with frequent entries into the corners, where they spent extended periods. These mice also regularly crossed the central area. In contrast, MPTP-induced mice displayed sparse trajectories, prolonged immobility, and significant reductions in total movement distance and mean speed, indicating successful induction of motor deficits. Compared with the MPTP group, mice in the MPTP + YCFS1, MPTP + YCFS3, MPTP + YCFS5, and MPTP + YLB groups demonstrated expanded movement ranges as shown by trajectory plots and heatmaps, as well as significantly elevated total distance and mean speed (Figure 1B–E). These results suggest that both *Lactobacillus reuteri* DSM 17938 or its supernatant alleviated MPTP-induced motor deficits in PD mice.

### 3.2. Lactobacillus reuteri DSM 17938 or Its Supernatant Alleviate MPTP-Induced Neuronal Death

**Figure 2:**
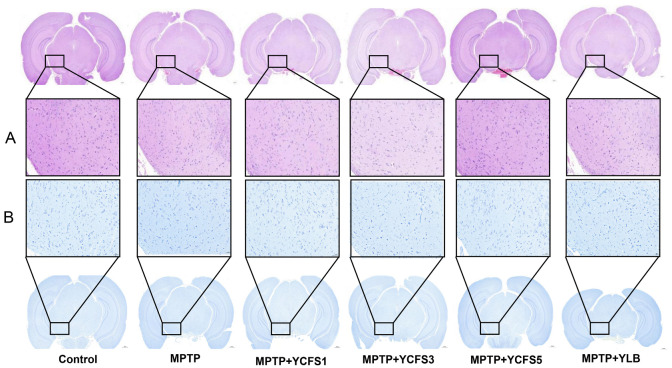
Effects of *Lactobacillus reuteri* DSM 17938 or supernatant treatment on H&E and Nissl staining in the substantia nigra of PD mice, with n = 3 biological replicates per group. (**A**) Representative H&E-stained sections from each group. (**B**) Representative Nissl-stained sections from each group. Scale bars = 500 μm (applies to all panels; original magnification at ×20).

**Figure 3:**
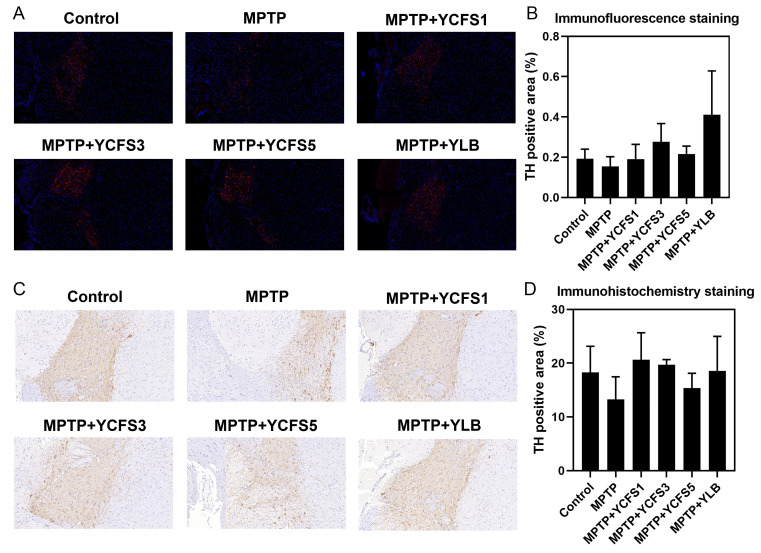
Tyrosine hydroxylase (TH) immunofluorescence and immunohistochemistry in the substantia nigra of Parkinson’s disease (PD) mice following treatment with *Lactobacillus reuteri* DSM 17938 or its culture supernatant, with n = 3 biological replicates per group. (**A**) Representative immunofluorescence micrographs of brain sections from each group. (**B**) Quantification of TH-immunoreactive area (%), presented as a bar graph, from the immunofluorescence sections. (**C**) Representative immunohistochemical micrographs of brain sections from each group. (**D**) Quantification of TH-positive area (%), presented as a bar graph, from the immunohistochemical sections. Data are expressed as means ± SEM and analyzed via one-way ANOVA.

To investigate the neuroprotective effects of *Lactobacillus reuteri* DSM 17938 or its supernatant on MPTP-induced PD mice, we performed HE staining, Nissl staining, immunofluorescence staining and immunohistochemical staining for TH on the SN region of mouse brains. Results of HE staining revealed that the SN of control mice contained a relatively high number of neurons, with no observed necrosis or infiltration. In contrast, the MPTP group exhibited a decreased number of neurons, accompanied by shrunken cell bodies, irregular morphology, poorly defined nuclear boundaries, and deepened staining. Relative to the MPTP group, the MPTP + YCFS1, MPTP + YCFS3, MPTP + YCFS5, and MPTP + YLB groups showed an increase in positive neurons and a reduction in shrunken cells within the SN (Figure 2A). Nissl staining indicated that neurons in the control group displayed a plump morphology, whereas those in the MPTP group appeared withered and shrunken, with reduced cell volume and irregular shape. Compared to the MPTP group, the MPTP + YCFS1, MPTP + YCFS3, MPTP + YCFS5, and MPTP + YLB groups all demonstrated ameliorated neuronal morphology and an increased number of neurons in the SN (Figure 2B). In contrast, all treated groups, such as MPTP + YLB, MPTP + YCFS1, MPTP + YCFS3, and MPTP + YCFS5, showed increasing trends in the SN region relative to the MPTP group (Figure 3A,B). Quantitative analysis of the TH-positive cell area in corresponding regions on immunofluorescence-stained sections is shown in Figure 3B. Compared with the MPTP-treated group, the TH-positive rates in the MPTP + YLB, MPTP + YCFS1, MPTP + YCFS3, and MPTP + YCFS5 groups increased by 23.51%, 79.35%, 40.06%, and 166.95%, respectively. Similarly, morphometric analysis of the TH-positive cell area in the same regions on immunohistochemistry-stained sections, also presented in Figure 3D, revealed increases of 55.42%, 48.42%, 15.89%, and 39.83% relative to the MPTP group; however, none of these differences reached statistical significance. Collectively, these results indicate that treatment with *Lactobacillus reuteri* DSM 17938 or its supernatant ameliorated MPTP-induced neuronal damage and increased the numbers of neuronal number in the SN of mice.

### 3.3. Lactobacillus reuteri DSM 17938 or Its Supernatant Alleviate MPTP-Induced Oxidative Stress and Inflammatory

**Figure 4:**
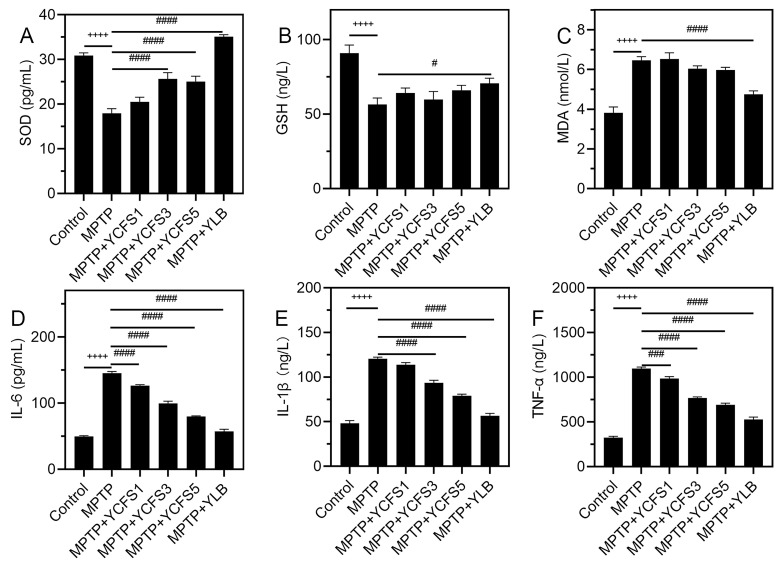
Effects of *Lactobacillus reuteri* DSM 17938 or supernatant treatment on serum oxidative stress parameters and inflammatory markers in PD mice, with n = 3 biological replicates per group. (**A**) The content of SOD in serum (pg/mL). (**B**) The content of GSH in serum (ng/mL). (**C**) The content of MDA in serum (nmol/mL). (**D**) The content of IL-6 in serum (pg/mL). (**E**) The content of IL-1β in serum (ng/mL). (**F**) The content of TNF-α in serum (ng/mL). Where the ++++ represents that the difference between MPTP and control was significant, and *p* < 0.001. The #, ###, and #### represents that the difference between MPTP + YCFS1, MPTP + YCFS3, MPTP + YCFS5, and MPTP + YLB with MPTP was significant, and #: *p* < 0.05, ###: *p* < 0.001 and ####: *p* < 0.0001; nonsignificant differences were not labeled. Data are expressed as means ± SEM and analyzed via one-way ANOVA.

To investigate the alleviating effects of *Lactobacillus reuteri* DSM 17938 or its supernatant on MPTP-induced oxidative damage and inflammatory responses in a PD mouse model, the serum contents of SOD (superoxide dismutase), MDA (malondialdehyde), GSH (glutathione), TNF-α (tumor necrosis factor-α), IL-1β (interleukin-1β), and IL-6 (interleukin-6) were measured. Compared with control, MPTP treatment significantly (*p* < 0.0001) decreased serum SOD and GSH levels, and significantly increasing the content of IL-6, IL-1β, TNF-α and MDA in serum (Figure 4A–F). These results indicated that MPTP induction caused oxidative damage and triggered an inflammatory response in the mice. Compared with MPTP, the levels of SOD and GSH was increased in the MPTP + YCFS1, MPTP + YCFS3, MPTP + YCFS5, and MPTP + YLB groups. The content of SOD was increased by 14.4%, 42.8% (*p* < 0.0001), 39.6% (*p* < 0.0001), and 95.7% (*p* < 0.0001), respectively. The content of GSH was increased by 13.6%, 5.87%, 16.8%, and 23.7% (*p* < 0.05), respectively (Figure 1A,B). Compared to the MPTP, serum MDA content decreased in the MPTP + YCFS3, MPTP + YCFS5, and MPTP + YLB, with a significant decrease (*p* < 0.0001) observed in the MPTP + YLB (Figure 4C). Serum levels of IL-6, IL-1β, and TNF-α were reduced in the MPTP + YCFS1, MPTP + YCFS3, MPTP + YCFS5, and MPTP + YLB compared to the MPTP. The reductions in IL-6 were 13.1%, 31.5%, 45.0%, and 60.7% (*p* < 0.0001), respectively. The reductions in IL-1β were 5.6%, 22.5% (*p* < 0.0001), 34.4% (*p* < 0.0001), and 53.1% (*p* < 0.0001), respectively. The reductions in TNF-α were 10.1% (*p* < 0.001), 29.8% (*p* < 0.0001), 36.9% (*p* < 0.0001), and 51.9% (*p* < 0.0001), respectively (Figure 4D–F). These results demonstrate that *Lactobacillus reuteri* DSM 17938 or its supernatant effectively alleviated MPTP-induced peripheral oxidative damage and systemic inflammatory responses in PD mice. Moreover, the MPTP + YLB exhibited superior antioxidant and anti-inflammatory effects compared to the MPTP + YCFS1, MPTP + YCFS3, and MPTP + YCFS5.

### 3.4. Lactobacillus reuteri DSM 17938 or Its Supernatant Modulates the Fecal Microbiota Composition

**Figure 5:**
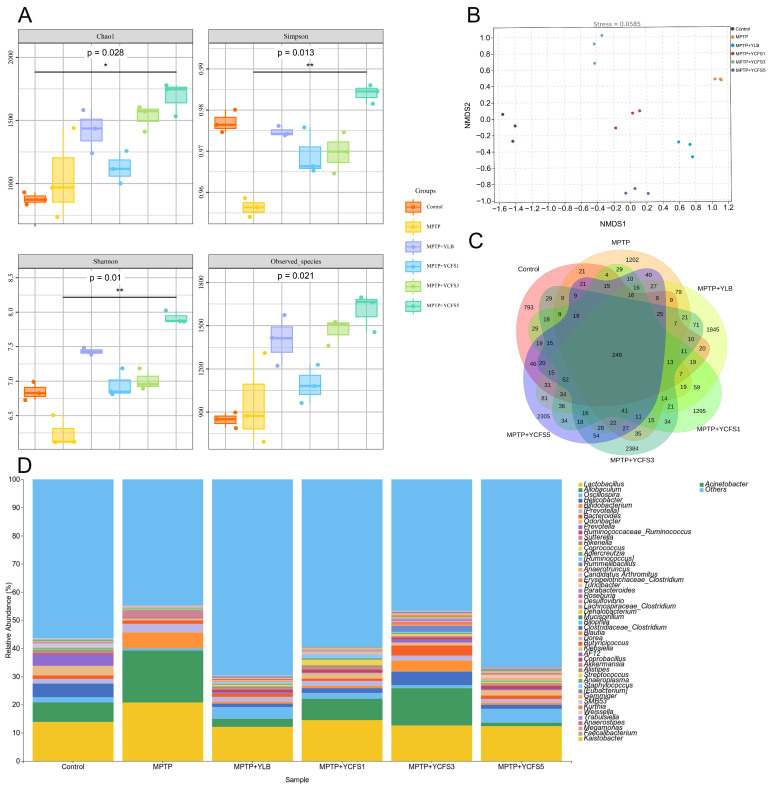
Effects of *Lactobacillus reuteri* DSM 17938 or its supernatant treatments on MPTP-induced PD mice fecal microbiomes, with n = 3 biological replicates per group. (**A**) Alpha diversity indices (Chao1, Observed species, Shannon, Simpson) for each group, presented as mean ± SEM and assessed by the Kruskal–Wallis test followed by Dunn’s post hoc test for pairwise comparisons among all groups. (**B**) NMDS plot based on Bray–Curtis distance for beta diversity; statistical significance of group separation was assessed by PERMANOVA (999 permutations). (**C**) Venn diagram showing the number of unique and shared ASVs across all groups. (**D**) Relative abundance (%) of the dominant microbial genera in each group.

To investigate how *Lactobacillus reuteri* DSM 17938 or its supernatant affect the gut microbiota of PD mice, we performed 16S-rRNA sequencing on fecal samples from each group. Alpha diversity analysis showed that, compared with control group, MPTP group exhibited an increased Chao1 index and observed species but a decreased Shannon index and Simpson index. All treatment groups (MPTP + YCFS1, MPTP + YCFS3, MPTP + YCFS5, and MPTP + YLB) showed increases in Shannon index and Simpson index compared with MPTP group, suggesting that MPTP induction increased gut microbiota richness but reduced diversity, and that treatments modulated the microbiota and ameliorated microbes diversity in PD mice (Figure 5A). NMDS analysis of beta diversity, based on the Bray–Curtis distance metric, revealed distinct compositional structures among the six groups (Figure 5B). The PERMANOVA (with 999 permutations) revealed highly significant differences in microbial community composition across all groups (*p* = 0.001), indicating that the experimental interventions collectively exerted a substantial impact on the gut microbiota architecture. Samples from the control, MPTP + YCFS1, MPTP + YCFS3, MPTP + YCFS5, and MPTP + YLB groups clustered closely, suggesting low intra-group variation, while different groups showed significant separation. Hierarchical clustering analysis revealed that the MPTP + YCFS3 group exhibited the highest similarity in microbes composition with control group, and the MPTP group showed considerable differences in microbiota composition compared to the other treatment groups (Figure S1A). The MPTP and control groups exhibited the greatest distance, indicating substantial differences in gut microbiota composition. However, administration of *Lactobacillus reuteri* DSM 17938 or its supernatant significantly mitigated these differences. Venn diagram analysis showed total ASV numbers of 1041, 1450, 1543, 2632, 2550, and 2093 for the control, MPTP, MPTP + YCFS1, MPTP + YCFS3, MPTP + YCFS5, and MPTP + YLB groups, respectively, with 248 ASV shared among all six groups (Figure 5C). Taxonomic analysis showed that the top four dominant microbes in the control group were *Oscillospira*, *Lactobacillus*, *Allobaculum*, and *Helicobacter*. Compared with control, the MPTP group showed higher *Lactobacillus* and *Allobaculum* but decreased relative abundances of *Oscillospira* and *Helicobacter*. In contrast, all treatment groups showed decreased relative abundances of *Lactobacillus* and *Allobaculum* and increased relative abundances of *Oscillospira* compared with MPTP group (Figure 5D and Figure S1B).

### 3.5. Fecal Untargeted Metabolomics Analysis of Lactobacillus reuteri DSM 17938 or Its Supernatant Treatment on MPTP-Induced PD Mice

**Figure 6:**
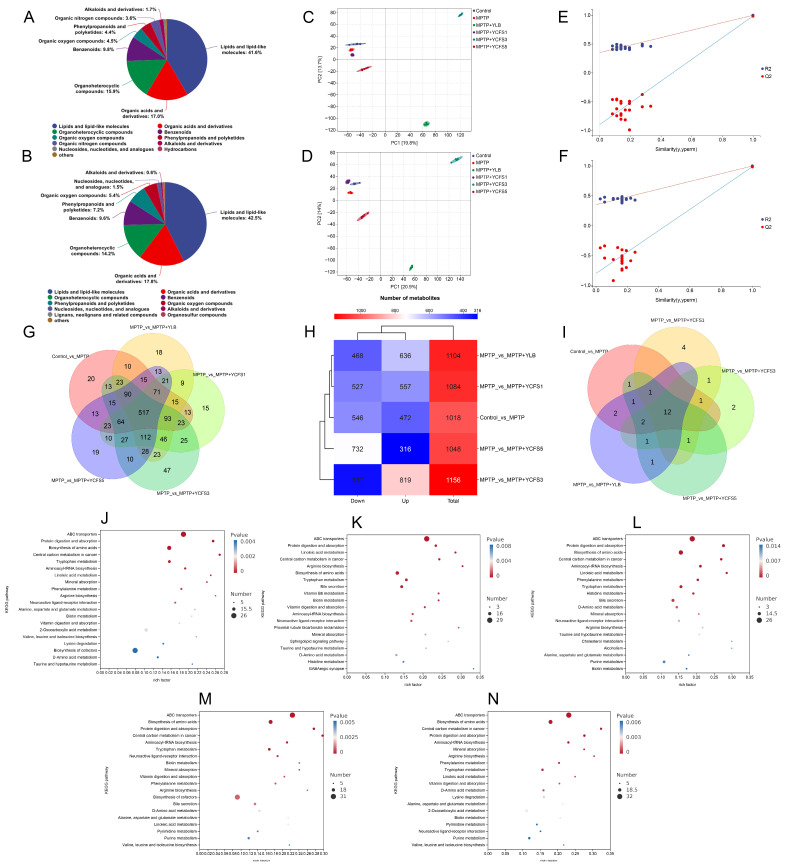
Fecal metabolomics analysis of MPTP-induced PD mice treated with *Lactobacillus reuteri* DSM 17938 or its supernatant, with n = 6 biological replicates per group. (**A**) The metabolite identification and analysis in the POS model. (**B**) The metabolite identification and analysis in the NEG model. (**C**) PCA in the POS model. (**D**) PCA in the NEG model. (**E**) PLS-DA permutation test plots in POS model. (**F**) PLS-DA permutation test plots in NEG model. (**G**) A Venn diagram of the fecal differential metabolites in each group. (**H**) Heat map showing the number of differential metabolites in each group. (**I**) A Venn diagram of the KEGG enrichment of fecal differential metabolites in each group (top 20 pathways based on *p*-value). (**J**) Control_vs_MPTP group. (**K**) MPTP_vs_MPTP + YCFS1. (**L**) MPTP_vs_MPTP + YCFS3. (**M**) MPTP_vs_MPTP + YCFS5. (**N**) MPTP_vs_MPTP + YLB.

This study investigated the impact of *Lactobacillus reuteri* DSM 17938 or its supernatant on the profile of gut microbiota-derived fecal metabolites in PD mice. Untargeted metabolomics was used to analyze the differential metabolites in fecal samples from each group. All QC sample correlation coefficients were above 0.9 (Figure S2). After processing, the identified metabolites were grouped by chemical taxonomy. In both modes, the most abundant metabolites were lipids and lipid-like, organic acids benzenoids, heterocyclics, and derivatives. (Figure 6A,B). The principal component analysis (PCA) results indicated significant differences in fecal metabolites among the six groups, with PC1 = 19.8% and PC2 = 13.7% in the POS model and PC1 = 20.9% and PC2 = 14.0% in the NEG model (Figure 6C,D). Similarly, PLS-DA results revealed that R^2^ values (R^2^X = 0.591, R^2^Y = 0.999 in POS models; R^2^X = 0.604, R^2^Y = 0.999 in NEG models) were greater than Q^2^ values (Q^2^ = 0.985 in both models) in both the POS and NEG models, suggesting a well-suited model for subsequent data analysis, as further confirmed by permutation testing (n = 200 permutations) with Q^2^ intercepts below zero in both ionization modes, indicating no overfitting and reliable predictive ability (Figure 6E,F). The number of differential metabolites between different groups was analyzed. We obtained 1018 differential metabolites between control and MPTP, and 1084, 1156, 1048, and 1104 when comparing MPTP with MPTP + YCFS1, MPTP + YCFS3, MPTP + YCFS5, and MPTP + YLB, respectively (Figure 6G). To further clarify the changes in fecal-related metabolites ameliorated by *Lactobacillus reuteri* DSM 17938 or its supernatant treatment in MPTP-induced PD mice, differential metabolites between control_vs_MPTP, MPTP_vs_MPTP + YCFS1, MPTP_vs_MPTP + YCFS3, MPTP_vs_MPTP + YCFS5, and MPTP_vs_MPTP + YLB groups were comparatively analyzed. There are 517 differential metabolites were identified in control_vs_MPTP, MPTP_vs_MPTP + YCFS1, MPTP_vs_MPTP + YCFS3, MPTP_vs_MPTP + YCFS5, and MPTP_vs_MPTP + YLB (Figure 6H). The mechanisms associated with *Lactobacillus reuteri* DSM 17938 or its supernatant ameliorating PD were revealed by KEGG pathways analysis. There are a total of 12 pathways were common to the control_vs_MPTP, MPTP_vs_MPTP + YCFS1, MPTP_vs_MPTP + YCFS3, MPTP_vs_MPTP + YCFS5, and MPTP_vs_MPTP + YLB groups, including ABC transporters, mineral absorption, linoleic acid metabolism, protein digestion and absorption, arginine biosynthesis, central carbon metabolism in cancer, aminoacyl-tRNA biosynthesis, tryptophan metabolism, biotin metabolism, biosynthesis of amino acids, neuroactive ligand-receptor interaction, and D-amino acid metabolism (Figure 6I–N).

### 3.6. The Effects of Lactobacillus reuteri DSM 17938 or Its Supernatant Treatment on Tryptophan Metabolism in Fecal Samples of MPTP-Induced PD Mice

**Figure 7:**
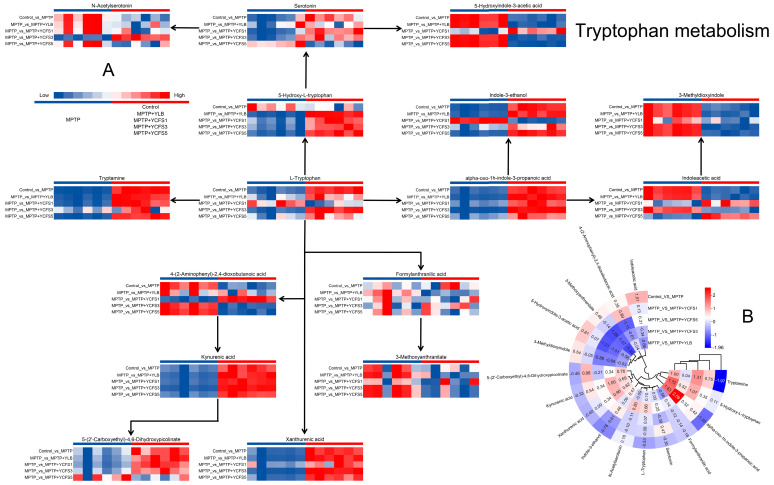
Response network of fecal differential metabolites in tryptophan metabolism pathways. (**A**) Network heat map, where red represents up-regulation and blue represents down-regulation. (**B**) Heatmap of log2FC values of fecal differential metabolites.

Targeting tryptophan metabolism is a possible novel therapeutic approach for PD treatment [28]. Accordingly, we conducted in-depth analysis of specific changes in metabolites in the tryptophan metabolic pathway in each group of mouse fecal samples. Changes in fecal tryptophan metabolites paralleled gut microbial dynamics. Except for being used in protein synthesis in organisms, tryptophan mainly follows three metabolic pathways: the kynurenine pathway, tryptamine pathway, and indole pathway [29]. The result of this study found that 16 differentially expressed metabolites were mapped to these three major pathways of tryptophan metabolism. Using metabolomics data, we constructed a network map of fecal tryptophan metabolism based on the KEGG pathway map of metabolite linkages, allowing visualization of interactions among different metabolites in the pathway. The analysis of the differentially expressed metabolites among experimental groups demonstrated that in the control_vs_MPTP group, 9 metabolites had been down-regulated and 7 were up-regulated. Within the tryptamine pathway, tryptamine is a polyamine synthesized from tryptophan. The results indicate that PD mice had reduced putatively annotated relative abundances of tryptamine metabolites in the gut, while treatment with *Lactobacillus reuteri* DSM 17938 or its supernatant restored tryptamine putatively annotated relative abundances. Serotonin, a crucial neuroactive substance, is synthesized from 5-Hydroxy-L-tryptophan. Compared with control group, MPTP-induced mice displayed increased putatively annotated relative abundances of 5-Hydroxy-L-tryptophan but decreased putatively annotated relative abundances of serotonin in fecal samples, indicating impaired conversion of 5-Hydroxy-L-tryptophan to serotonin due to altered gut microbiota in MPTP-induced PD mice. Treatment with *Lactobacillus reuteri* DSM 17938 or its supernatant restored fecal serotonin putatively annotated relative abundances. In the indole pathway, alpha-oxo-1H-indole-3-propanoic acid is a substrate for the synthesis of indoleacetic acid and indole-3-ethanol. MPTP-induced PD led to decreased putatively annotated relative abundances of alpha-oxo-1H-indole-3-propanoic acid and indole-3-ethanol but increased indoleacetic acid putatively annotated relative abundances. Treatment with *Lactobacillus reuteri* DSM 17938 or its supernatant ameliorated tryptophan metabolism by gut microbiota, elevating the putatively annotated relative abundances of alpha-oxo-1H-indole-3-propanoic acid and indole-3-ethanol in PD mice fecal samples. Compared to the MPTP, MPTP + YLB treatment reduced the putatively annotated relative abundance of indoleacetic acid in fecal samples. Relative to controls, MPTP induction also lowered fecal putatively annotated relative abundances of kynurenic acid and xanthurenic acid within the kynurenine pathway. However, the treatment of MPTP + YLB, MPTP + YCFS1, MPTP + YCFS3, and MPTP + YCFS5 showed increased putatively annotated relative abundances of kynurenic acid and xanthurenic acid compared to the MPTP (Figure 7A,B and Figure S3). In summary, compared with control, MPTP group exhibited reduced putatively annotated relative abundances of the neuroactive substances like serotonin, kynurenic acid, and xanthurenic acid in fecal samples, along with elevated putatively annotated relative abundances of pro-inflammatory substances such as indoleacetic acid. Treatment with *Lactobacillus reuteri* DSM 17938 or its supernatant restored the fecal putatively annotated relative abundances of serotonin, kynurenic acid, and xanthurenic acid, while reducing the putatively annotated relative abundances of 3-Methyldioxyindole and 5-Hydroxyindole-3-acetic acid. The results suggest that treatment with *Lactobacillus reuteri* DSM 17938 or its supernatant can modulate tryptophan metabolism in the gut microbiota, ameliorate the metabolic disruption of tryptophan in PD mice, and restore the putatively annotated relative abundances of neuroactive substances in both intestinal and fecal samples.

### 3.7. Identification and Analysis of Tryptophan Metabolism Associated Microbiota

**Figure 8:**
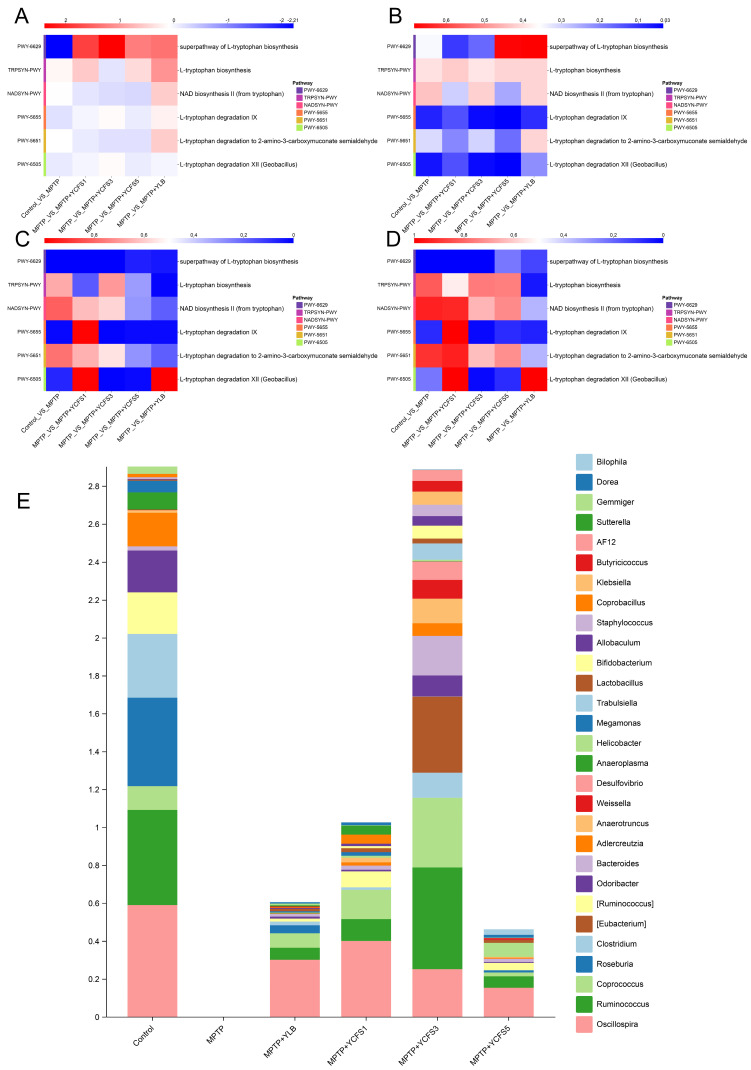
Analysis of tryptophan metabolism-associated pathways in microbiota, with n = 3 biological replicates per group. (**A**) Heatmap showing the Log_2_FC of pathways across different groups. (**B**) Heatmap showing the standard error of pathways across different groups. (**C**) Heatmap showing the *p*-values of pathways across different groups. (**D**) Heatmap showing the adjusted *p*-values of pathways across different groups. (**E**) Microbial composition of pathway PWY-6629.

To investigate the predicted functional pathways microbiota associated with tryptophan metabolism in this study, PICRUSt2 was employed to perform statistical analysis of differentially enriched predicted metabolic pathways, predicted pathway variations, and species composition. The statistical analysis of predicted metabolic pathways revealed higher abundance of microbiota involved in the biosynthesis of amino acids, nucleosides and nucleotides, and cofactors, prosthetic groups, electron carriers, and vitamins. (Figure S4). Compared with the control group, the predicted levels of microbiota related to amino acid biosynthesis decreased in the MPTP group but increased in the MPTP + YCFS1, MPTP + YCFS3, MPTP + YCFS5, and MPTP + YLB groups. This indicates that *Lactobacillus reuteri* DSM 17938 or its supernatant promote the accumulation of amino acid biosynthesis-associated microbiota in PD mice by modulating the gut microbiome. Analysis of differentially enriched metabolic pathways identified six predicted pathways related to tryptophan metabolism: the super-pathway of L-tryptophan biosynthesis (PWY-6629), L-tryptophan degradation IX (PWY-5655), L-tryptophan degradation XII (Geobacillus) (PWY-6505), L-tryptophan degradation to 2-amino-3-carboxymuconate semialdehyde (PWY-5651), L-tryptophan degradation IX (TRPSYN-PWY), and NAD biosynthesis II (from tryptophan) (NADSYN-PWY) (Figure 8A–D). The predicted abundance of microbiota associated with pathway PWY-6629 was significantly reduced in MPTP group compared with control group but markedly increased in the MPTP + YCFS1, MPTP + YCFS3, MPTP + YCFS5, and MPTP + YLB groups. Further analysis of the species composition of pathway PWY-6629 revealed that 29 bacterial genera are involved in tryptophan metabolism, with Oscillospira, Ruminococcus, Coprococcus, Roseburia, and Clostridium exhibiting higher abundance across all groups (Figure 8E).

## 4. Discussion

PD is a neurodegenerative disorder whose global prevalence is steadily increasing, highlighting an urgent need for effective interventions that also have minimal adverse effects [30]. Substantial evidence indicates gut microbiota alterations and consequent tryptophan metabolism disruptions play pivotal roles in the pathogenesis of PD [31]. These findings suggest that modulating the gut microbiome to promote the accumulation of neuroactive tryptophan metabolites could represent a promising therapeutic strategy. Consequently, probiotic supplementation is considered a promising approach for PD management. *Lactobacillus reuteri* DSM 17938, a well-documented probiotic, is widely used in the intervention of several disorders. However, the mechanism whereby *Lactobacillus reuteri* DSM 17938 and its supernatant ameliorates PD through modulating gut microbiota and tryptophan metabolism is still not fully understood.

*Lactobacillus reuteri* has exhibited therapeutic potential in ameliorating a range of pathological conditions, including neuroinflammation, diabetes-induced cardiomyopathy [32], liver failure [33], depression [34], and Parkinson’s disease [35]. PD is commonly linked with motor dysfunction, and in this study oral administration of *Lactobacillus reuteri* DSM 17938 or its supernatant significantly alleviated motor deficits in PD mice, as evidenced by enhanced exploratory behavior, along with increased total movement distance and mean speed. These results are consistent with prior studies demonstrating that probiotic supplementation attenuated MPTP-induced motor impairments in PD models [36]. A central histopathological feature in PD is the loss of dopaminergic neurons within the substantia nigra (SN) [37]. Tyrosine hydroxylase (TH) is the rate-limiting enzyme for dopamine synthesis and serves as a well-established indicator of dopaminergic integrity. Its expression level in the SN also positively correlates with neuronal survival [38]. Previous research has indicated that *Lactobacillus reuteri* DSM 17938 can mitigate MPTP-induced neuronal loss [11]. Correspondingly, our results show that MPTP administration resulted in significant neuronal damage and depletion in the SN, accompanied by decreased TH expression. Intervention with either *Lactobacillus reuteri* DSM 17938 or its supernatant attenuated neuronal injury, showed a tendency to increase TH expression, and reduced dopaminergic degeneration, with whole bacterial treatment producing more substantial protective effects compared to its supernatant alone.

The contribution of neuroinflammation to PD progression and the therapeutic potential of anti-inflammatory approaches are both underscored by clinical evidence showing elevated IL-6, IL-1β, and TNF-α levels in patients [39]. Concurrently, MDA is widely recognized as a key biomarker of oxidative stress in PD, and its serum levels reflect the severity of systemic oxidative damage and treatment response [40]. Excessive reactive oxygen species promote lipid peroxidation, resulting in MDA formation. And MDA subsequently cross-links nucleic acids and proteins, thereby aggravating cellular injury [41]. Antioxidant enzymes and antioxidant compounds such as SOD and GSH counteract ROS accumulation, reduce MDA production, and mitigate oxidative damage. Thus, assessing circulating inflammatory and oxidative stress parameters offers valuable direct insight into the systemic protective mechanisms of treatments in MPTP-induced PD models [42]. Our findings reveal that both *Lactobacillus reuteri* DSM 17938 or its supernatant significantly attenuated serum levels of proinflammatory cytokines including IL-6, IL-1β and TNF-α, and decreased MDA content, while simultaneously raising SOD and GSH levels, indicating amelioration of peripheral inflammation and oxidative stress. These outcomes match a previous report in which *L. plantarum* treatment notably decreased IL-6, IL-1β, and TNF-α levels in the brain of PD mice and elevated antioxidant enzyme activity [43], suggesting that probiotic interventions may confer benefits at both peripheral and central levels. Collectively, these results indicate that *Lactobacillus reuteri* DSM 17938 and its supernatant administration confers strong anti-inflammatory and antioxidant effects in PD mice, with the whole bacterial treatment offering superior efficacy compared to the supernatant.

Increasing data demonstrates that PD is frequently related to shifts in gut microbiome structure and dysregulation of tryptophan metabolism in gut [13]. Consequently, modulating the gut microbiome to facilitate the production of neuroprotective tryptophan metabolites has gained recognition as a promising therapeutic strategy for PD management [44,45]. Notably, both PD patients and MPTP-induced PD models mice showed increased levels of *Lactobacillus* and *Allobaculum* at the genus level [43,46,47], a finding that aligns with the baseline observations of this study. However, following intervention with *Lactobacillus reuteri* DSM 17938 or its supernatant, we observed a reduction in the levels of *Lactobacillus* and *Allobaculum*, alongside a rise in the proportion of *Oscillospira* in the gut microbiota of PD mice. Previous reports have demonstrated that probiotic supplementation can ameliorate gut dysbiosis in PD mice by enriching *Oscillospira* [48]. Similarly, previous studies have shown that probiotic treatment can ameliorate PD by increasing the abundance of *Oscillospira* and other bacterial genera in the gut of PD mice, which corroborates the observations from our study [49]. Taken together, these results suggest that MPTP induction may lead to pronounced shifts in the gut microbial community PD mice, while treatment with *Lactobacillus reuteri* DSM 17938 or its supernatant appears to partially normalize the microbial composition.

Tryptophan metabolites are established as key mediators of gut–brain communication [50]. Non-targeted metabolomic profiling of fecal samples revealed that the tryptophan metabolic pathway was among the most significantly enriched pathways in KEGG analysis across the different treatment groups. This observation is consistent with a large body of prior study underscoring the central role of gut microbiota regulation of tryptophan metabolism in neurodegenerative processes [51,52]. Based on this, we carried out a comprehensive examination and visualization of DAMs within the tryptophan metabolic pathway among the experimental groups. The results indicate that treatment with *Lactobacillus reuteri* DSM 17938 or its supernatant promoted the accumulation of several neuroprotective tryptophan metabolites in the gut, including serotonin, xanthurenic acid, kynurenic acid, and alpha-oxo-1H-indole-3-propanoic acid, while reducing the putatively annotated relative abundances of compounds such as 3-methyldioxyindole and 5-hydroxyindole-3-acetic acid. It is well established that the gut serves as the major site of serotonin production in humans, where it acts as a key signaling mediator modulating gastrointestinal movement and secretory activity [53]. Neuroprotective tryptophan metabolites from the kynurenine (xanthurenic and kynurenic acids) and indole (alpha-oxo-1H-indole-3-propanoic acid) routes are boosted in the gut to effectively counteract PD pathology [54]. Previous studies have reported that dysregulation of this pathway in PD patients leads to the accumulation of potentially harmful neuroactive intermediates [52], these observations are consistent with the current data. The above study showed that treatment with *Lactobacillus reuteri* DSM 17938 or its supernatant significantly increased the accumulation of neuroprotective active tryptophan metabolites in the gut of PD mice, and the accumulation of neuroactive metabolites derived from the tryptophan-kynurenine pathway in fecal samples was closely associated with the observed gut microbes shifts. The accumulation of neuroactive metabolites derived from the tryptophan metabolites in fecal samples was closely associated with the observed gut microbes shifts. To explore the microbiota related to tryptophan metabolism, predicted metabolic pathway analysis of the microbiota was performed using PICRUSt2 [27]. The results identified several microbes taxa associated with the predicted superpathway of L-tryptophan biosynthesis (PWY-6629), including *Oscillospira*, *Ruminococcus*, *Coprococcus*, *Roseburia*, and *Clostridium* which exhibited relatively higher predicted abundance across all experimental groups. These data indicate that modulating the gut microbiota through supplementation with *Lactobacillus reuteri* DSM 17938 or its supernatant to preserve the abundance of tryptophan-metabolizing microbes such as *Oscillospira*, *Ruminococcus*, and *Coprococcus* may be an important approach for ameliorating PD.

Intact Lactobacillus reuteri DSM 17938 outperformed its supernatant in peripheral serum antioxidant/anti-inflammatory capacity and open-field locomotor activity. However, the underlying mechanisms remain unclear. Several limitations exist: bacterial colonization or in situ metabolic turnover was not measured, supernatant bioactive components were not identified, and the effects of lyophilization degradation and transient supernatant action were not validated, rendering the above interpretations speculative; single strain, single-sex mice and subacute MPTP models cannot fully recapitulate progressive clinical PD pathology; only serum inflammatory/oxidative markers and open-field locomotion were assessed, lacking brain tissue profiling and broader behavioral tests; missing vehicle controls (pH-neutralized supernatant or processed MRS broth) obscure solvent effects; PICRUSt2 predictions from 16S data cannot substitute shotgun multi-omics; insufficient fecal samples prevented microbe-tryptophan metabolite correlation analyses, and key tryptophan derivatives lacked LC-MS/MS absolute quantification; histological sample size was consistent with previous MPTP studies, but some assays had small sample sizes, and larger cohorts would enhance statistical power; although blinding was applied in behavioral tests, randomization and blinding reporting remain not fully detailed, and the lack of treatment-only groups limits data interpretation. Future work will compare whole-cell and supernatant treatments, identify functional metabolites through metabolomics, and test combinatorial therapies in chronic, sex-balanced animal models including primates. Fecal microbiota transplantation will verify the neuroprotective roles of *Oscillospira* and *Ruminococcus*, while tryptophan metabolite supplementation and integrated network pharmacology with molecular docking will clarify anti-PD mechanisms.

## 5. Conclusions

In conclusion, *Lactobacillus reuteri* DSM 17938 and its supernatant both confer neuroprotection in MPTP-induced PD mice by modulating gut microbiota and tryptophan metabolism, with live bacteria showing superior efficacy. The neuroprotective effect is characterized by restoration of *Oscillospira* abundance, accumulation of neuroactive tryptophan metabolites (kynurenic acid, xanthurenic acid, and serotonin), and suppression of pro-inflammatory indoleacetic acid. This protection reflects an intra-pathway shift within the kynurenine route from neurotoxic to neuroprotective branches, rather than inter-pathway diversion to indole metabolism. The greater potency of whole-cell treatment likely arises from sustained gut colonization and continuous metabolic activity, whereas supernatant effects are more transient. PICRUSt2 inference identified *Oscillospira*, *Ruminococcus*, and *Coprococcus* as putative tryptophan-metabolizing taxa, suggesting that preserving these microbes may be therapeutically relevant. Together, these findings establish that *Lactobacillus reuteri* DSM 17938 alleviates PD through microbiota-tryptophan metabolic axis modulation, and supports targeting specific tryptophan-metabolizing bacteria or their metabolites as a promising strategy for PD intervention.

## Data Availability

The raw data of 16S rRNA high-throughput sequencing was uploaded to the Sequence Read Archive (SRA) database of NCBI, accession number: PRJNA1345351 (https://www.ncbi.nlm.nih.gov/sra/PRJNA1345351, accessed on 15 July 2026), and the raw data of the untargeted metabolomics has been uploaded to the Metabolights database, accession number: MTBLS13169 (https://www.ebi.ac.uk/metabolights/MTBLS13169, accessed on 15 July 2026). The remaining data are available upon request from the corresponding author or the first author.

## Supplementary Materials

The following supporting information can be downloaded at: https://www.mdpi.com/article/10.3390/antiox15070882/s1. Figure S1: Effects of *Lactobacillus reuteri* DSM17938 and its supernatant treatments on MPTP-induced PD mouse fecal microbiomes. (A) Hierarchical clustering analysis of gut microbiota composition. (B) Heat map of gut microbiota composition at the genus level. Figure S2: Quality control analysis of metabolomics data. (A) Total ion chromatogram in the POS model. (B) Total ion chromatogram in the NEG model. (C) Quality control (QC) sample correlation analysis in POS model. (D) Quality control (QC) sample correlation analysis in NEG model. Figure S3: Heat maps of P values and VIP values of metabolites in tryptophan metabolism pathways. (A) Heat map of P values. (B) Heat map of VIP values. Figure S4: Microbial metabolic pathway analysis. The abundance (in KO/PWY/COG per million) or the count of functional pathways/categories; the vertical axis lists the specific pathways or categories at the secondary hierarchical level. The rightmost column indicates the corresponding primary-level classification for each entry.

## References

[1] Nie S., Wang J., Deng Y., Ye Z., Ge Y. (2022). Inflammatory microbes and genes as potential biomarkers of Parkinson’s disease. npj Biofilms Microbiomes.

[2] Cacabelos R. (2017). Parkinson’s Disease: From Pathogenesis to Pharmacogenomics. Int. J. Mol. Sci..

[3] Khan M.S., Nasiripour S., Bopassa J.C. (2025). Parkinson Disease Signaling Pathways, Molecular Mechanisms, and Potential Therapeutic Strategies: A Comprehensive Review. Int. J. Mol. Sci..

[4] Ebadpour N., Mahmoudi M., Kheder R.K., Abavisani M., Baridjavadi Z., Abdollahi N., Esmaeili S.-A. (2024). From mitochondrial dysfunction to neuroinflammation in Parkinson‘s disease: Pathogenesis and mitochondrial therapeutic approaches. Int. Immunopharmacol..

[5] Fan H.X., Sheng S., Zhang F. (2022). New hope for Parkinson’s disease treatment: Targeting gut microbiota. CNS Neurosci. Ther..

[6] Cryan J.F., Dinan T.G. (2012). Mind-altering microorganisms: The impact of the gut microbiota on brain and behaviour. Nat. Rev. Neurosci..

[7] Romano S., Savva G.M., Bedarf J.R., Charles I.G., Hildebrand F., Narbad A. (2021). Meta-analysis of the Parkinson’s disease gut microbiome suggests alterations linked to intestinal inflammation. NPJ Park. Dis..

[8] Mirzaei H., Sedighi S., Kouchaki E., Barati E., Dadgostar E., Aschner M., Tamtaji O.R. (2022). Probiotics and the Treatment of Parkinson’s Disease: An Update. Cell. Mol. Neurobiol..

[9] Mu Q., Tavella V.J., Luo X.M. (2018). Role of *Lactobacillus reuteri* in Human Health and Diseases. Front. Microbiol..

[10] Salminen S., Collado M.C., Endo A., Hill C., Lebeer S., Quigley E.M.M., Sanders M.E., Shamir R., Swann J.R., Szajewska H. (2021). The International Scientific Association of Probiotics and Prebiotics (ISAPP) consensus statement on the definition and scope of postbiotics. Nat. Rev. Gastroenterol. Hepatol..

[11] Dong X., Yang T., Jin Z. (2025). *Lactobacillus reuteri*-derived γ-amino butyric acid alleviates MPTP-induced Parkinson’s disease through inhibiting ferroptosis via the AKT-GSK3β-GPX4 axis. NPJ Park. Dis..

[12] Cen Q., Cui Y., Feng J., Zhu L., Wei J., Wang L., Chang C., Pang R., Wang J., Zhang A. (2025). *Limosilactobacillus reuteri* DSM17938 Attenuates Neuroinflammatory Responses After Spinal Cord Injury by Modulating Tryptophan Metabolism. Probiotics Antimicrob. Proteins.

[13] Wang W., Jiang S., Xu C., Tang L., Liang Y., Zhao Y., Zhu G. (2022). Interactions between gut microbiota and Parkinson’s disease: The role of microbiota-derived amino acid metabolism. Front. Aging Neurosci..

[14] Lai Z., Gong F. (2024). Protective Effects of *Lactobacillus reuteri* on Intestinal Barrier Function in a Mouse Model of Neonatal Necrotizing Enterocolitis. Am. J. Perinatol..

[15] Yi H., Wang L., Xiong Y., Wang Z., Qiu Y., Wen X., Jiang Z., Yang X., Ma X. (2018). *Lactobacillus reuteri* LR1 Improved Expression of Genes of Tight Junction Proteins via the MLCK Pathway in IPEC-1 Cells during Infection with Enterotoxigenic Escherichia coli K88. Mediat. Inflamm..

[16] Platten M., Nollen E.A.A., Röhrig U.F., Fallarino F., Opitz C.A. (2019). Tryptophan metabolism as a common therapeutic target in cancer, neurodegeneration and beyond. Nat. Rev. Drug Discov..

[17] Gao K., Mu C.L., Farzi A., Zhu W.Y. (2020). Tryptophan Metabolism: A Link Between the Gut Microbiota and Brain. Adv. Nutr..

[18] Alkhalaf L.M., Ryan K.S. (2015). Biosynthetic Manipulation of Tryptophan in Bacteria: Pathways and Mechanisms. Chem. Biol..

[19] Liu N., Sun S., Wang P., Sun Y., Hu Q., Wang X. (2021). The Mechanism of Secretion and Metabolism of Gut-Derived 5-Hydroxytryptamine. Int. J. Mol. Sci..

[20] Hestad K., Alexander J., Rootwelt H., Aaseth J.O. (2022). The Role of Tryptophan Dysmetabolism and Quinolinic Acid in Depressive and Neurodegenerative Diseases. Biomolecules.

[21] Török N., Tanaka M., Vécsei L. (2020). Searching for Peripheral Biomarkers in Neurodegenerative Diseases: The Tryptophan-Kynurenine Metabolic Pathway. Int. J. Mol. Sci..

[22] Heilman P.L., Wang E.W., Lewis M.M., Krzyzanowski S., Capan C.D., Burmeister A.R., Du G., Escobar Galvis M.L., Brundin P., Huang X. (2020). Tryptophan Metabolites Are Associated With Symptoms and Nigral Pathology in Parkinson’s Disease. Mov. Disord..

[23] Szabó N., Kincses Z.T., Toldi J., Vécsei L. (2011). Altered tryptophan metabolism in Parkinson’s disease: A possible novel therapeutic approach. J. Neurol. Sci..

[24] Xue C., Li G., Zheng Q., Gu X., Shi Q., Su Y., Chu Q., Yuan X., Bao Z., Lu J. (2023). Tryptophan metabolism in health and disease. Cell Metab..

[25] Liao J.-F., Cheng Y.-F., You S.-T., Kuo W.-C., Huang C.-W., Chiou J.-J., Hsu C.-C., Hsieh-Li H.-M., Wang S., Tsai Y.-C. (2020). Lactobacillus plantarum PS128 alleviates neurodegenerative progression in 1-methyl-4-phenyl-1,2,3,6-tetrahydropyridine-induced mouse models of Parkinson’s disease. Brain Behav. Immun..

[26] Callahan B.J., McMurdie P.J., Rosen M.J., Han A.W., Johnson A.J.A., Holmes S.P. (2016). DADA2: High-resolution sample inference from Illumina amplicon data. Nat. Methods.

[27] Douglas G.M., Maffei V.J., Zaneveld J.R., Yurgel S.N., Brown J.R., Taylor C.M., Huttenhower C., Langille M.G.I. (2020). PICRUSt2 for prediction of metagenome functions. Nat. Biotechnol..

[28] Agus A., Planchais J., Sokol H. (2018). Gut Microbiota Regulation of Tryptophan Metabolism in Health and Disease. Cell Host Microbe.

[29] Holeček M. (2026). Serotonin, Kynurenine, and Indole Pathways of Tryptophan Metabolism in Humans in Health and Disease. Nutrients.

[30] Willis A.W., Roberts E., Beck J.C., Fiske B., Ross W., Savica R., Van Den Eeden S.K., Tanner C.M., Marras C., Alcalay R. (2022). Incidence of Parkinson disease in North America. NPJ Park. Dis..

[31] Iwaniak P., Owe-Larsson M., Urbańska E.M. (2024). Microbiota, Tryptophan and Aryl Hydrocarbon Receptors as the Target Triad in Parkinson‘s Disease—A Narrative Review. Int. J. Mol. Sci..

[32] Chiang C.J., Tsai B.C., Lu T.L., Chao Y.P., Day C.H., Ho T.J., Wang P.N., Lin S.C., Padma V.V., Kuo W.W. (2021). Diabetes-induced cardiomyopathy is ameliorated by heat-killed *Lactobacillus reuteri* GMNL-263 in diabetic rats via the repression of the toll-like receptor 4 pathway. Eur. J. Nutr..

[33] Jiang H., Yan R., Wang K., Wang Q., Chen X., Chen L., Li L., Lv L. (2021). *Lactobacillus reuteri* DSM 17938 alleviates d-galactosamine-induced liver failure in rats. Biomed. Pharmacother..

[34] Han S.K., Kim J.K., Joo M.K., Lee K.E., Han S.W., Kim D.H. (2020). *Lactobacillus reuteri* NK33 and Bifidobacterium adolescentis NK98 Alleviate Escherichia coli-Induced depression and Gut Dysbiosis in Mice. J. Microbiol. Biotechnol..

[35] Aktas B., Aslim B., Ozdemir D.A. (2024). A neurotherapeutic approach with Lacticaseibacillus rhamnosus E9 on gut microbiota and intestinal barrier in MPTP-induced mouse model of Parkinson’s disease. Sci. Rep..

[36] Tchung A., Even A., Trudeau L.-É. (2026). Cell-autonomous and non-cell-autonomous drivers of dopamine neuron vulnerability in Parkinson‘s disease. Trends Neurosci..

[37] Nagatsu T., Nagatsu I. (2016). Tyrosine hydroxylase (TH), its cofactor tetrahydrobiopterin (BH4), other catecholamine-related enzymes, and their human genes in relation to the drug and gene therapies of Parkinson’s disease (PD): Historical overview and future prospects. J. Neural Transm..

[38] Zhou Z.D., Tan E.K. (2022). The role of tyrosine hydroxylase–dopamine pathway in Parkinson’s disease pathogenesis. Cell. Mol. Life Sci..

[39] Shateri S., Khatami S.H., Toutounchi A.H., Rajaei S., Mahdavi M., Baram S.M., Shahidi G.-A., Habibi A.H., Aghamollaii V., Ghlichnia B. (2023). Plasma cytokines profile in patients with Alzheimer’s and Parkinson‘s Disease: A comparative study in terms of inflammation. Int. J. Neurosci..

[40] Cheng J., Wang F., Yu D.F., Wu P.F., Chen J.G. (2011). The cytotoxic mechanism of malondialdehyde and protective effect of carnosine via protein cross-linking/mitochondrial dysfunction/reactive oxygen species/MAPK pathway in neurons. Eur. J. Pharmacol..

[41] Liu T., Kong X., Qiao J., Wei J. (2025). Decoding Parkinson’s Disease: The interplay of cell death pathways, oxidative stress, and therapeutic innovations. Redox Biol..

[42] Xia Y., Zhang C., Yu L., Zhang Q., Narbad A., Chen W., Zhai Q., Tian F. (2025). Tryptophan metabolism and the gut-brain axis: Focus on specific gut microbial genera. J. Future Foods.

[43] Wang L., Zhao Z., Zhao L., Zhao Y., Yang G., Wang C., Gao L., Niu C., Li S. (2022). Lactobacillus plantarum DP189 Reduces α-SYN Aggravation in MPTP-Induced Parkinson’s Disease Mice via Regulating Oxidative Damage, Inflammation, and Gut Microbiota Disorder. J. Agric. Food Chem..

[44] Takahashi K., Nishiwaki H., Ito M., Iwaoka K., Takahashi K., Suzuki Y., Taguchi K., Yamahara K., Tsuboi Y., Kashihara K. (2022). Altered gut microbiota in Parkinson’s disease patients with motor complications. Park. Relat. Disord..

[45] Cryan J.F., O‘Riordan K.J., Cowan C.S.M., Sandhu K.V., Bastiaanssen T.F.S., Boehme M., Codagnone M.G., Cussotto S., Fulling C., Golubeva A.V. (2019). The Microbiota-Gut-Brain Axis. Physiol. Rev..

[46] Tsai S.-T., Lai Z.-L., Lu M.-K., Walther-Antonio M., Hsu Y.-Y., Chao D.-S., Chen C.-Y., Vettleson-Trutza S., Kuo J.S., Yang C.-C. (2025). Elevated *Lactobacillus salivarius* and genus *Akkermansia* in fecal samples of Taiwanese patients with Parkinson’s disease and diabetes mellitus. Sci. Rep..

[47] Jo S., Kang W., Hwang Y.S., Lee S.H., Park K.W., Kim M.S., Lee H.J., Yoon S.S., Lee S.T., Chu K. (2022). Oral and gut dysbiosis leads to functional alterations in Parkinson’s disease. NPJ Park. Dis..

[48] Yue M., Wei J., Chen W., Hong D., Chen T., Fang X. (2022). Neurotrophic role of the next-generation probiotic strain *L. lactis* MG1363-pMG36e-GLP-1 on Parkinson’s disease via inhibiting ferroptosis. Nutrients.

[49] Andreozzi V., Cuoco S., Balestrieri M., Fierro F., Ferrara N., Erro R., Di Filippo M., Barbella G., Memoli M.C., Silvestri A. (2024). Synbiotic supplementation may globally improve non-motor symptoms in patients with stable Parkinson’s disease: Results from an open label single-arm study. Sci. Rep..

[50] Fathi M., Vakili K., Yaghoobpoor S., Tavasol A., Jazi K., Hajibeygi R., Shool S., Sodeifian F., Klegeris A., McElhinney A. (2022). Dynamic changes in metabolites of the kynurenine pathway in Alzheimer’s disease, Parkinson’s disease, and Huntington’s disease: A systematic Review and meta-analysis. Front. Immunol..

[51] Zhou Y., Chen Y., He H., Peng M., Zeng M., Sun H. (2023). The role of the indoles in the microbiota-gut-brain axis and potential therapeutic targets: A focus on human neurological and neuropsychiatric diseases. Neuropharmacology.

[52] Lovelace M.D., Varney B., Sundaram G., Lennon M.J., Lim C.K., Jacobs K., Guillemin G.J., Brew B.J. (2017). Recent evidence for an expanded role of the kynurenine pathway of tryptophan metabolism in neurological diseases. Neuropharmacology.

[53] Fan X., Xiao Z., Chen Y., Yang H., Diao M., Hu W., Wang S. (2025). Interactions between gut microbiota and Parkinson‘s disease: The role of tryptophan metabolism. Cell Commun. Signal..

[54] Shajib M.S., Baranov A., Khan W.I. (2017). Diverse Effects of Gut-Derived Serotonin in Intestinal Inflammation. ACS Chem. Neurosci..

