# Supplementary material for: Lactobacillus reuteri DSM 17938 and Its Supernatant Ameliorate Parkinson’s Disease in Association with Modulation of Gut Microbiota and Its Tryptophan Metabolism
Source: Antioxidants (Basel). 2026 Jul 16;15(7):882. doi: 10.3390/antiox15070882 (PMC13406045; doi:10.3390/antiox15070882)
Supplement: Supplementary file 1 [file antioxidants-15-00882-s001.zip › antioxidants-4373932-supplementary.pdf]

Supporting information to:

***Lactobacillus reuteri* DSM 17938 and its supernatant ameliorate Parkinson's disease in association with modulation of gut microbiota and its tryptophan metabolism**

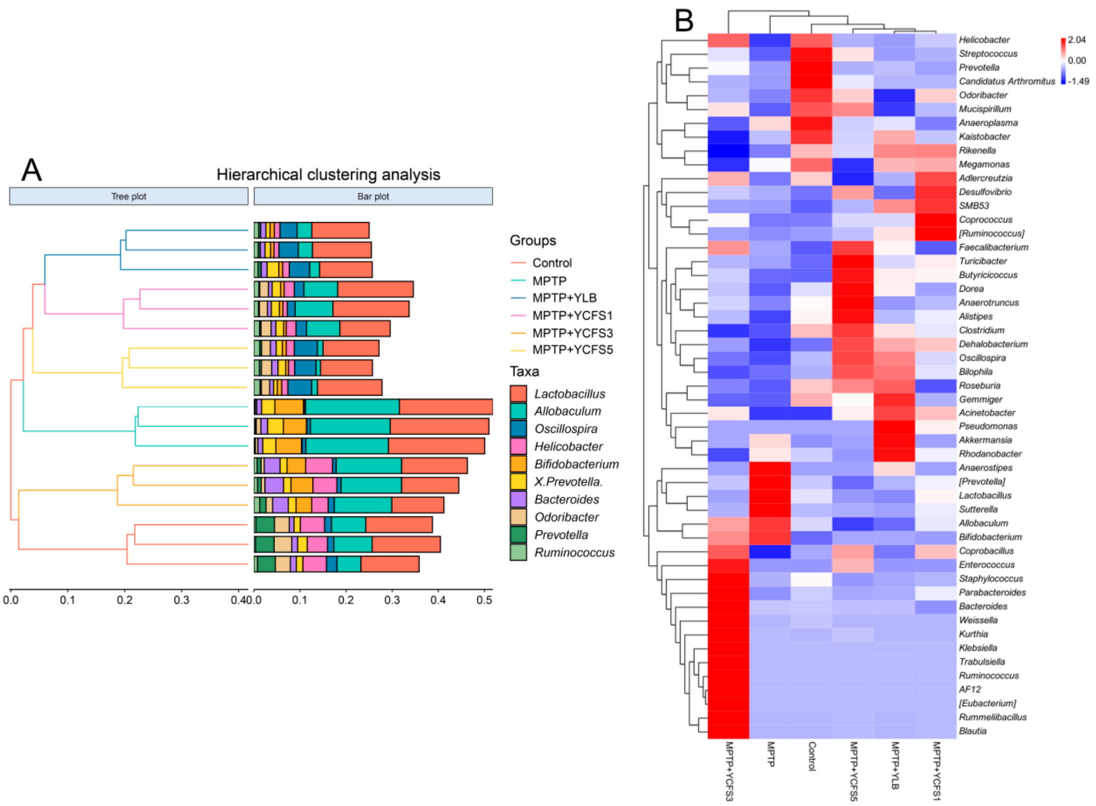

Figure S1. Effects of *Lactobacillus reuteri* DSM17938 and its supernatant treatments on MPTP-induced PD mouse fecal microbiomes. (A) Hierarchical clustering analysis of gut microbiota composition. (B) Heat map of gut microbiota composition at the genus level.

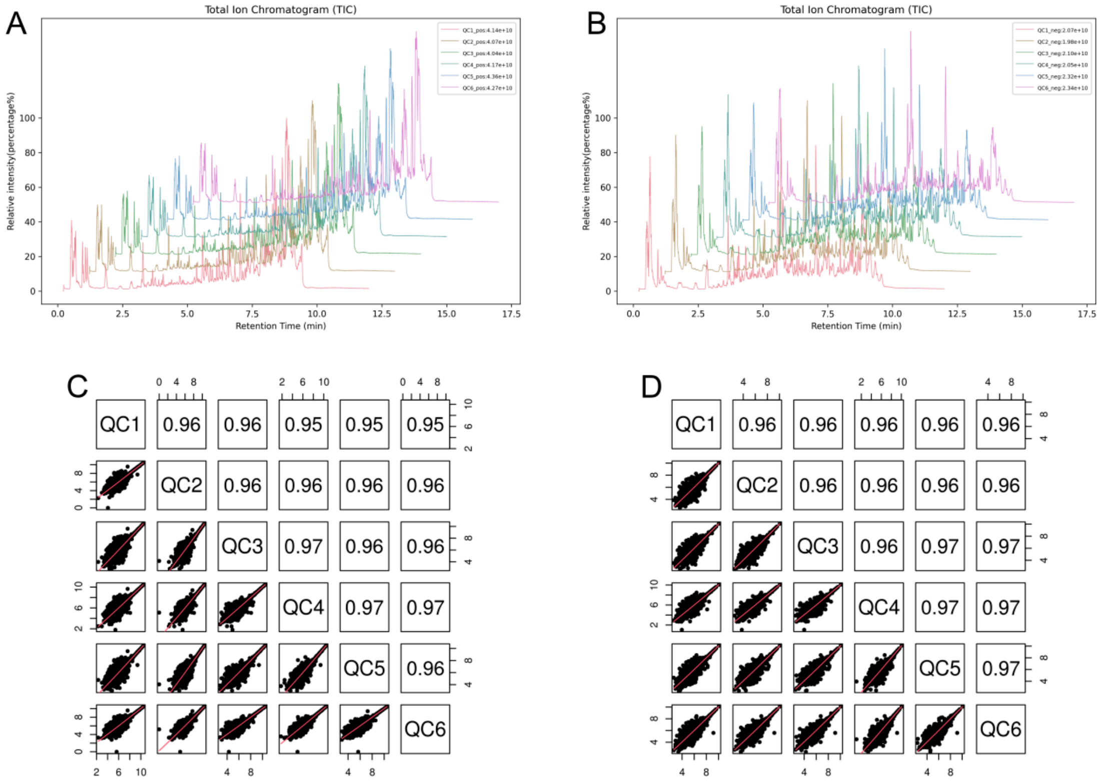

Figure S2. Quality control analysis of metabolomics data. (A) Total ion chromatogram in the POS model. (B) Total ion chromatogram in the NEG model. (C) Quality control (QC) sample correlation analysis in POS model. (D) Quality control (QC) sample correlation analysis in NEG model.

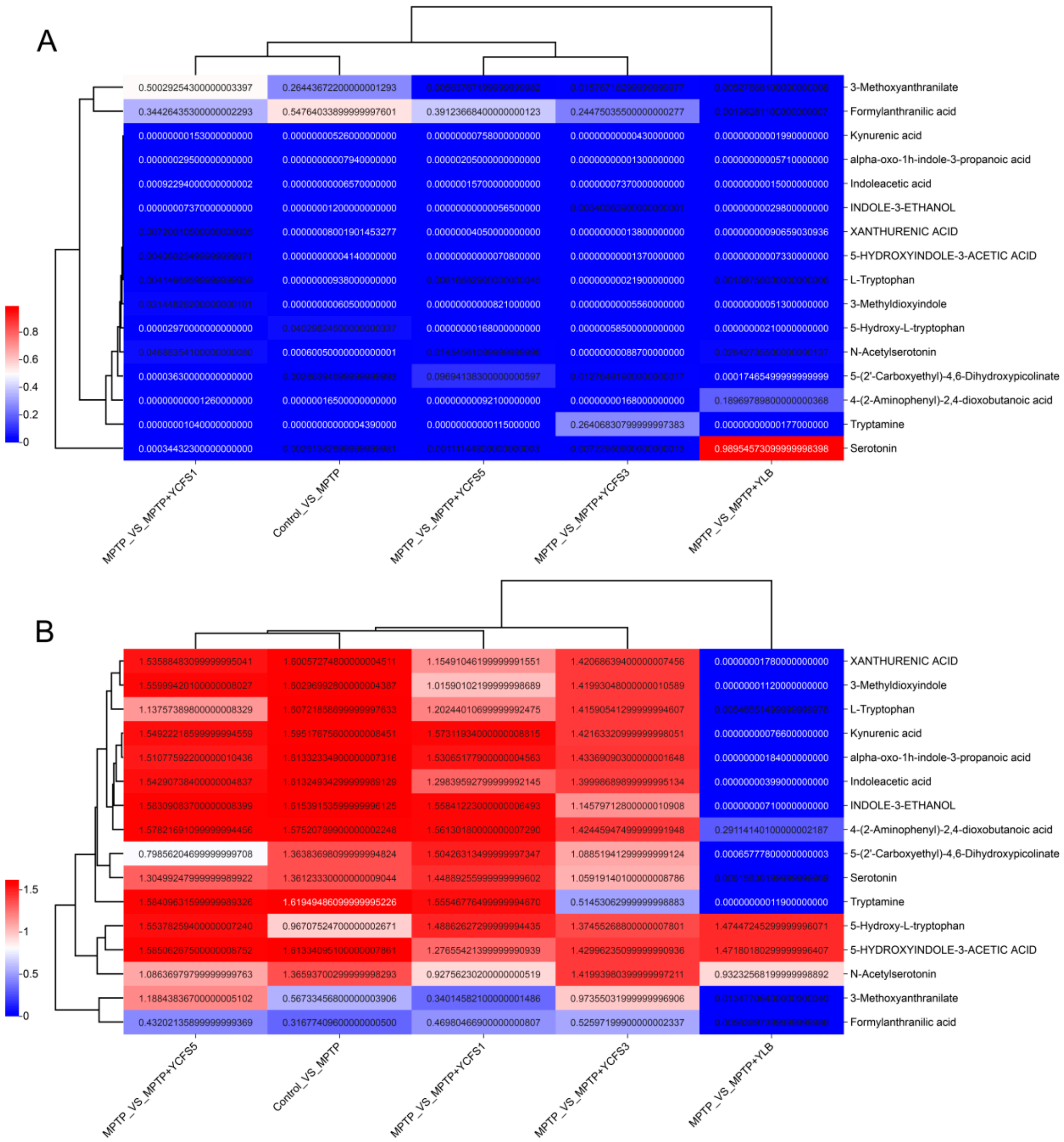

Figure S3. Heat maps of P values and VIP values of metabolites in tryptophan metabolism pathways. (A) Heat map of P values. (B) Heat map of VIP values.

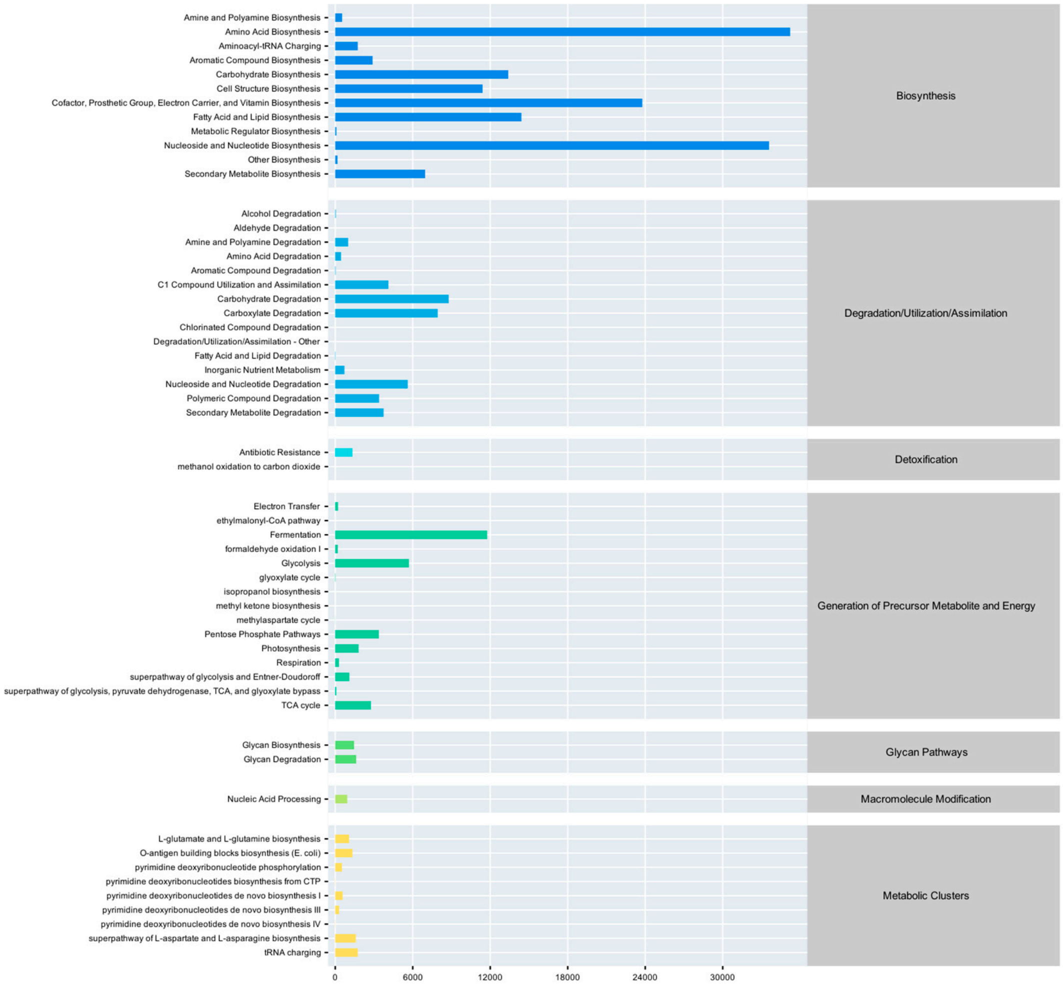

Figure S4. Microbial metabolic pathway analysis. The abundance (in KO/PWY/COG per million) or the count of functional pathways/categories; the vertical axis lists the specific pathways or categories at the secondary hierarchical level. The rightmost column indicates the corresponding primary-level classification for each entry.
